# Evaluating learning formats in international collaborative nursing education on professional agency in perioperative sustainability: protocol for a mixed-methods, randomised pilot study

**DOI:** 10.1136/bmjopen-2026-124998

**Published:** 2026-11-11

**Authors:** Marie Therese Crafoord, Karianne Barlein, Eva Torbjörnsson, Magnhild Vikan, Ida Andrea Macintosh Kvalvik, Ami Fagerdahl, Stein Ove Danielsen

**Affiliations:** 1 Department of Clinical Science and Education, Södersjukhuset – KI SÖS, Karolinska Institute, Stockholm, Sweden; 2 Acute and Critically Ill Patients, Institute of Nursing and Health Promotion, Oslo Metropolitan University Faculty of Health Sciences, Oslo, Norway; 3 Department of Perioperative and Intensive Care, Södersjukhuset, Stockholm, Sweden; 4 Department of Nursing and Health Promotion, Oslo Metropolitan University Faculty of Health Sciences, Oslo, Norway

**Keywords:** Perioperative Nursing, Operating Rooms, MEDICAL EDUCATION & TRAINING

## Abstract

**Abstract:**

**Introduction:**

Perioperative care is a highly resource-intensive component of healthcare, with operating theatres consuming substantial energy and generating considerable waste and emissions. Sustainable perioperative practice is therefore essential to reduce environmental impact. However, there is limited understanding of how competencies in sustainable practice can be developed within operating theatre nursing education. In particular, little attention has been given to how educational interventions shape students’ professional agency in relation to sustainability. Collaborative Online International Learning (COIL) offers a pedagogical approach that supports reflective and intercultural learning and may offer opportunities to foster professional agency for sustainability. This protocol describes a mixed-methods, randomised, feasibility pilot study that aims to assess the feasibility and acceptability of implementing and evaluating two learning formats in international collaborative learning in theatre nursing education, while also generating preliminary insights into how these formats may influence students’ reflective work and expressions of professional agency.

**Methods and analysis:**

A 90-minute synchronous online seminar focusing on sustainable theatre nursing practice across perioperative care phases will be delivered as part of the curriculum to operating theatre nursing students at Karolinska Institutet (Sweden) and Oslo Metropolitan University (Norway). Students will be randomly allocated (1:1) to one of two formats: (1) phase-specific COIL, in which small groups focus on a single perioperative phase, followed by whole-group sharing across phases, with documentation in a digital space; or (2) cross-phase COIL, in which small groups address all phases, document their work using a self-selected format and subsequently engage in whole-group comparison. A pragmatic sample of 24–36 students is anticipated. Primary outcomes are feasibility and acceptability indicators, including consent rates, questionnaire completion and follow-up rates, intervention fidelity, participant engagement and the feasibility of repeated questionnaire administration. Qualitative data, including collaborative outputs and observer field notes, will be analysed using reflexive thematic analysis informed by a conceptual model of professional agency and sustainability competence. Comparative analyses will explore differences in the complexity of students’ expressions of professional agency across learning formats. Quantitative data will include students’ perceptions of intercultural collaboration and learning, as well as attitudes towards sustainability in the operating theatre, assessed before the seminar and 1 week later. Because questionnaire responses are anonymous and cannot be linked at the individual level, baseline and follow-up data will be analysed as independent aggregate datasets. Quantitative findings will therefore be interpreted descriptively and will not be used to assess individual-level changes over time. Integration of qualitative and quantitative findings will be conducted at the interpretation stage using a triangulation approach.

**Ethics and dissemination:**

The study has been approved by the Norwegian Agency for Shared Services in Education and Research (reference number 266740). In Sweden, the study was reviewed by the Swedish Ethical Review Authority (reference number 2026-00252-01), which concluded that the study did not require formal ethical approval under the Swedish Ethical Review Act and issued an advisory opinion. Participation in the research component is voluntary, and informed consent will be obtained. All data will be de-identified prior to analysis. Findings will be disseminated through peer-reviewed publications and conference presentations and will inform the design of a future randomised controlled trial (RCT) of an educational intervention aimed at supporting sustainable practice in operating theatre nursing education.

**Study registration:**

Open Science Framework (OSF; 10.17605/OSF.IO/YMF6V). Protocol version: 2.0 (23 September 2026), incorporating clarifications made prior to intervention delivery and outcome data collection.

**STRENGTHS AND LIMITATIONS OF THIS STUDY**

- Use of a theoretically informed and transparent analytical framework integrating professional agency and sustainability competencies enables structured and nuanced analysis.
- Mixed-methods design, combining multiple qualitative and quantitative data sources, supports a comprehensive exploration of how different learning formats shape reflective processes.
- The study is not powered for statistical inference, and voluntary participation in post-session data collection may introduce response bias.
- Analysis focuses on articulated reflections rather than observed behaviour, limiting conclusions about translation to clinical practice; potential cross-contamination between groups and lack of blinding may also influence findings, and transferability may be context-dependent.

## Introduction

 The healthcare sector is a major contributor to global greenhouse gas emissions, with surgical care accounting for a substantial share of this impact.^1^ Operating theatre departments are key drivers of hospitals’ environmental burden, due to their disproportionate energy consumption and significant generation of waste and emissions.^2–5^ These impacts are largely associated with energy-intensive heating, ventilation and air-conditioning systems, as well as the extensive use of single-use instruments and consumables.^3 5^ Nevertheless, evidence suggests that targeted sustainability initiatives can reduce emissions and costs without compromising patient safety.^6^ Theatre departments therefore have a responsibility to contribute to the United Nations Sustainable Development Goals, particularly those related to health (Goal 3), responsible consumption (Goal 12) and climate action (Goal 13).^6 7^

Sustainability in operating theatres has increasingly been examined in relation to healthcare professionals’ behaviours, attitudes and organisational constraints.^8 9^ This body of research highlights that advancing sustainable practice depends not only on technical solutions but also on professionals’ awareness, knowledge and the contexts in which they work. In Scandinavia, perioperative professionals recognise climate change as a pressing healthcare challenge but report uneven preparedness and limited organisational support.^10^ However, there remains limited understanding of how these competencies, particularly awareness, knowledge and context-sensitive judgement, are developed within postgraduate nursing education. In particular, the ways in which educational interventions can support the development of professional agency for sustainable perioperative practice have received little attention^8 9^

Both Karolinska Institutet (KI) and Oslo Metropolitan University (OsloMet) forefront sustainability and internationalisation in their Specialist Nursing Programmes in Operating Theatre Care, integrating sustainable healthcare competencies alongside global and intercultural perspectives in education. This is reflected in institutional strategies^11–13^ and regulatory frameworks that emphasise sustainable practice and global engagement such as the Norwegian Regulation on Education for Operating Theatre Nurses 2022, §2.^14^ Together, these priorities provide a foundation for a collaborative learning approach that combines sustainability with intercultural exchange.

The planned study described in this protocol is part of a larger, ongoing collaborative project between KI and OsloMet, aiming at strengthening how operating theatre nursing education prepares students to engage with sustainable practice. Within this collaboration, faculty from both institutions will jointly develop educational activities that seek to align sustainability knowledge with students’ own clinical observations and support their capacity to contribute to changes in practice. The overall project adopts a stepwise approach to examine feasibility and generate initial insights, followed by a larger evaluative study.

### Conceptual framework

An integrated conceptual framework has been outlined, drawing on sustainability education^15^ and professional agency^16^ to conceptualise support for operating theatre nursing students’ development of ethically informed, contextually responsive sustainable practice (figure 1). Sustainability education frameworks emphasise that professional action is not only technical but also shaped by values. This means that professionals need to critically reflect on what counts as responsible and ethically justified practice. A key part of this is the ability to engage in ethical reasoning and to balance competing values when making decisions about sustainable practice.^15^

**Figure 1:**
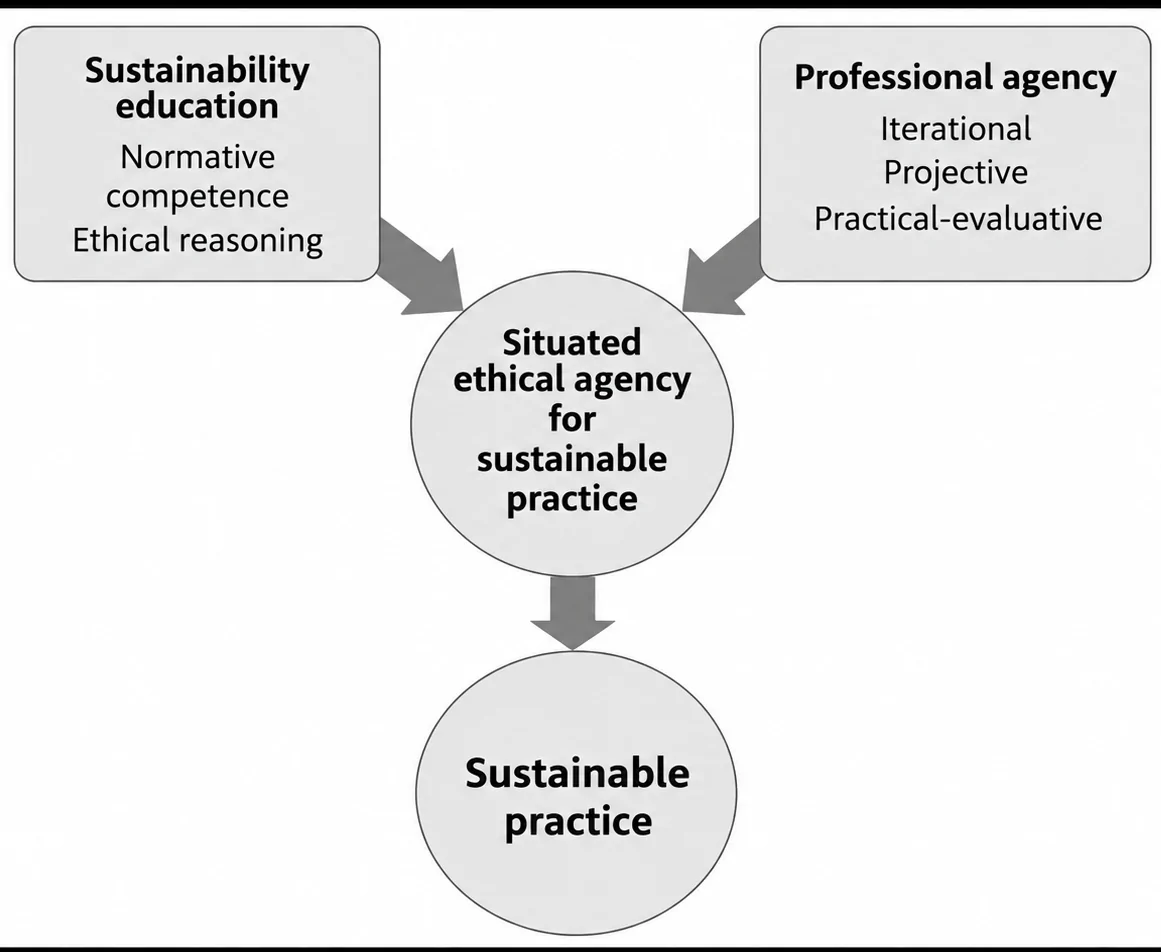
Conceptual framework for operating theatre nursing students’ development of ethically informed and contextually responsive sustainable practice. Developed by the authors, this model integrates sustainability education^15^ and professional agency^16^ to conceptualise support for operating theatre nursing students’ development of ethically informed and contextually responsive sustainable practice.

At the same time, professional agency has been described as multidimensional, context-dependent and relational, rather than as an individual trait. Agency is understood as emerging through the interaction between past experiences, future intentions and present, context-specific judgements. These three interconnected dimensions are described as iterational (drawing on past experiences and professional norms), projective (imagining and proposing future actions) and practical-evaluative (making judgements about what is possible and appropriate in a specific situation).^16^

Bringing these perspectives together is particularly important in the context of sustainability in healthcare, where professionals must navigate complex clinical realities shaped by uncertainty, competing priorities and systemic constraints, while simultaneously responding to ethical and normative demands. In this context, critical thinking is understood as a reflective process involving the application of knowledge, analysis and judgement in professional practice,^17^ forming a basis for developing contextually grounded and actionable professional agency.

Within nursing education, critical thinking is often described as a key process through which forms of judgement are developed and expressed.^18^ However, evidence on educational strategies to promote critical thinking remains inconsistent. A systematic review identified a wide range of approaches, including problem-based learning, simulation, concept mapping and other active learning strategies, with variable and sometimes conflicting results. Interventions that incorporated structured, collaborative and problem-oriented activities that required students to analyse clinical situations, justify decisions and reflect on practice tended to demonstrate more favourable patterns. At the same time, substantial heterogeneity in intervention design, duration and outcome measures limits conclusions about which approaches are most effective.^18^

Taken together, these findings suggest that the development of critical thinking,^18^ and by extension the articulation of professional agency,^16^ depends not only on the choice of pedagogical method but also on how learning activities are structured to engage students in analysis, evaluation and context-sensitive judgement. In particular, the organisation of interaction and opportunities to engage with multiple perspectives appear to influence the nature and form of students’ reflective work. This highlights a need for research that examines how different configurations of collaborative learning shape students’ reasoning rather than focusing solely on whether a given intervention is effective.

Collaborative Online International Learning (COIL)^19^ is an established pedagogical approach within higher education that enables students from different countries to engage in structured, technology-mediated collaboration as part of their formal curriculum. It is commonly conceptualised as a form of virtual exchange, characterised by shared or co-developed learning activities, collaborative tasks and guided interaction across cultural and geographical boundaries.^20^ COIL is grounded in experiential and collaborative learning principles and aims to integrate internationalisation into the curriculum in an inclusive and scalable way. Evidence indicates that COIL can support the development of intercultural competence, digital literacy and collaborative skills, while fostering students’ ability to engage with diverse perspectives.^20 21^

Intercultural learning outcomes from COIL are generally positive, particularly in cognitive and behavioural domains, although effects on attitudes are more variable and dependent on pedagogical design.^21^ COIL is not a standardised methodology but a flexible pedagogical approach that can be adapted to different disciplinary contexts and learning objectives. Effective implementation typically includes structured collaborative tasks, guided reflection and active facilitation by educators.^21 22^ As part of a COIL learning activity, operating theatre nursing students from both institutions will work together, focusing on sustainable theatre practice.^19^

The COIL activity planned for this pilot study is designed as an equal collaboration, with faculty from both institutions codeveloping and cofacilitating the session to ensure shared ownership and pedagogical alignment. The activity is designed to promote active learning through alignment of sustainability knowledge with students’ own clinical observations.

### Study rationale

Sustainable perioperative practice is essential to reduce the environmental impact of healthcare, yet translating sustainability principles into clinical practice requires not only knowledge but the capacity of healthcare professionals to critically evaluate, prioritise and enact change within complex organisational contexts. Preparing operating theatre nursing students for such practice therefore involves supporting their ability to engage in context-sensitive judgement and their motivation to initiate or influence changes in existing routines. This aligns with conceptualisations of professional agency as relational, situational and oriented towards practical action.

Educational strategies that aim to promote critical thinking may contribute to this development; however, existing evidence remains inconsistent, and it is unclear how different pedagogical designs support students’ capacity to move from reflection to actionable professional reasoning. In particular, there is limited understanding of how educational activities can be designed to foster the articulation of feasible and contextually grounded actions in practice.

Within a larger collaborative project between KI and OsloMet a learning activity has been developed to address these challenges by aligning sustainability knowledge with students’ clinical experiences and supporting reflective work on practice change. A pilot study is warranted to assess feasibility and to inform the design of a subsequent larger-scale evaluative study.

#### Comparator rationale

Two learning formats that differ primarily in how interaction and reflection are structured within the same educational activity will be compared. In the phase-specific COIL format, students are organised according to perioperative phases (pre-, intra- and post-operative) and engage in focused reflection within a single phase, supported by structured collaborative documentation in a digital workspace. In contrast, the cross-phase COIL format requires students to work across all perioperative phases and to jointly construct and document their reasoning using flexible, group-selected formats (eg, digital whiteboards or presentations), thereby emphasising negotiation of perspectives and collective prioritisation. Comparing these formats enables exploration of how different structures for collaboration and documentation shape the form and complexity of students’ reflective work.

### Aim

The primary aim of the pilot study is to assess the feasibility and acceptability of implementing and evaluating two pedagogically distinct learning formats within an international collaborative learning activity in theatre nursing education. This includes examining recruitment, participation, data collection procedures and indicators of engagement and fidelity. In addition, the study aims to assess the feasibility and acceptability of administering attitude questionnaires at two time points to measure students’ attitudes towards sustainability in the operating theatre. Analyses will be restricted to descriptive comparisons of aggregate group responses. Findings will inform the design of a future trial in which linked individual-level data may be collected.

As an exploratory aim, the study seeks to examine similarities and differences in operating theatre nursing students’ reflective work and expressions of professional agency in relation to sustainable perioperative practice across the two learning formats. Additionally, the study explores the students’ perceptions of intercultural collaboration, including their experiences of working in multicultural teams and reflecting on similarities and differences across healthcare contexts.

## Methods and analysis

### Study design

This study is designed as a randomised, convergent, parallel, mixed-methods, feasibility pilot study with two arms (1:1 allocation ratio). Qualitative and quantitative data will be collected concurrently, analysed separately and integrated at the interpretation stage. This design is aligned with established mixed-methods principles, where integration enables a more comprehensive understanding of complex phenomena than single-method approaches.^22^

The quantitative component will address feasibility outcomes and students’ perceptions of learning and intercultural collaboration. In addition, questionnaire data will be collected before the seminar and 1 week afterwards to describe patterns in attitudes towards sustainable perioperative practice at each time point and to assess the feasibility of repeated survey administration.

The qualitative component constitutes the main source of insight into the exploratory educational outcome and focuses on students’ reflective work as expressed through multiple data sources. This approach enables in-depth analysis of how professional agency is articulated in relation to sustainable perioperative practice.

Integration of qualitative and quantitative findings will be conducted using a triangulation approach at the interpretation stage, allowing complementary forms of evidence to be examined in relation to each other.^22 23^

### Setting

The study will be conducted within specialist nursing programmes in operating theatre care at KI, Sweden, and OsloMet, Norway. The intervention will be a part of the regular educational programme curriculum and delivered as a scheduled learning activity.

### Participants and recruitment

Participation in the educational activity is mandatory as part of the curriculum; however, participation in the research component is voluntary. All students scheduled to attend the seminar are eligible to participate in the research component. Students who are unable to attend the seminar due to illness or other reasons will be assigned an alternative written task to meet course requirements. All eligible students (approximately 22 from each institution) will receive an invitation to participate. Based on anticipated participation rates, a pragmatic sample of 24–36 students (12–18 per learning format) is considered feasible. Given the exploratory nature of this pilot study, no formal power calculation has been undertaken. Recruitment is planned to take place on 5 October 2026, prior to the seminar scheduled for the same day.

### Randomisation and allocation

Students will be randomised prior to the intervention in equal proportions (1:1) to one of the two learning formats. The randomisation sequence will be generated in advance by a member of the research team who is not involved in teaching, intervention delivery, outcome assessment or data collection (MV). Separate allocation sequences will be generated for each site using a web-based randomisation tool.^24^ To ensure balanced representation across sites, randomisation will be stratified by institution (KI and OsloMet) applying block randomisation with a block size of four. Students will not be informed of their allocation until the day of the intervention.

### Blinding

Blinding of study participants or researchers who also facilitate the seminars is not feasible due to the nature of the educational intervention. Participants will not be informed of the specific pedagogical differences between the study arms. To reduce the risk of confirmation bias, data analysis will be conducted collaboratively, and analytic decisions will be documented throughout the process.

There is a potential risk of contamination, as participants may communicate with peers after the intervention. This could influence their perceptions and responses to outcome measures. However, the seminar is conducted on the only scheduled on-campus day prior to the evaluation, after which students return to individual clinical placements, limiting opportunities for interaction. Taken together, these factors are expected to reduce, although not eliminate, the risk of cross-group influence.

### Intervention development

Prior to the present pilot study, a small-scale pre-pilot was conducted with four operating theatre nursing students recruited from the two participating institutions (n=2 from KI and n=2 from OsloMet). The purpose was to test procedures, logistics, learning materials, and delivery of the COIL activity. Feedback from participants was used to refine aspects of the intervention, including the organisation of preparatory materials, clarification of instructions, timing of activities, and facilitation procedures. In particular, contextual information about educational and clinical settings was moved from the synchronous session to asynchronous pre-seminar preparation to increase time available for interaction and reflection. Findings indicated that the use of English generally supported participation, and that the combination of English and shared Scandinavian languages facilitated communication and mutual understanding among participants.

### Educational intervention

The intervention comprises asynchronous preparatory activities, involving review of provided materials, followed by a 90-minute synchronous online COIL session delivered via videoconference (table 1). A dedicated course room for the COIL activity is created in the learning platform used at both participating institutions and made accessible to all students approximately 1 week prior to the intervention. Information about the activity and instructions for access are communicated via announcements within the students’ existing course rooms.

**Table 1:** Participant timeline

Study phase	Time	Activity
Randomisation and allocation	3 September 2026	Students are randomised 1:1 to one of the two learning formats, stratified by institution and assigned to pre-defined small groups. The allocation sequence is generated by a researcher not involved in teaching, intervention delivery, outcome assessment or data collection (MV). Students are not informed of their allocation.
Invitation and access to COIL course room	30 September 2026	Students receive an announcement in their current course room with information about the COIL activity scheduled for 5 October 2026 and instructions for accessing the dedicated COIL course room.
Pre-seminar preparation	5 October 2026, morning	Students access the COIL course room and individually review the preparatory materials, including the Intercollegiate Green Theatre Checklist^25^ and information about operating theatre nursing education and clinical practice in Sweden and Norway.
Study information and baseline assessment	5 October 2026, morning	The participant information sheet is available in the COIL course room. Students who choose to participate in the research provide consent by completing and submitting the baseline questionnaire on attitudes towards sustainable practice via a link in the course room.^27^
Common introductory session	5 October 2026, start of seminar	All students join the same online session and participate in a common introduction to perioperative sustainability, including ethical aspects of sustainable practice.
Ice-breaking activity	5 October 2026, during common session	Students join their pre-assigned small groups for an ice-breaking activity and then return to the common session. The same small-group composition is retained for the subsequent format-specific discussions.
Transition to allocated learning formats	5 October 2026, before scheduled break	Before the break, students receive a link to the online session corresponding to their pre-assigned allocation. The links are also posted in the COIL course room. Students are not provided with the format-specific discussion instructions until after the break.
Format-specific small-group discussion	5 October 2026, after scheduled break, 40 min	Students join their assigned online session, receive the format-specific discussion instructions and undertake the sustainability discussion in their pre-assigned small groups.
Plenary session	5 October 2026, 20 min	Students reconvene within their allocated learning format for a facilitated plenary discussion. Collaborative group artefacts produced during the seminar are collected for analysis.
Fidelity and adherence monitoring	5 October 2026, during seminar	Attendance, participation, adherence to the allocated learning format and seminar delivery are documented through session records and structured observations.
COIL learning evaluation	5 October 2026, immediately after seminar	Students are invited to complete the questionnaire on intercultural collaboration and learning.^29^ The questionnaire link is provided at the end of the seminar, posted in the COIL course room, and sent by email.
Follow-up assessment of attitudes towards sustainability	12 October 2026, 1 week after seminar	Students receive an email containing the participant information sheet and a link to the follow-up questionnaire on attitudes towards sustainable practice.^27^

COIL, Collaborative Online International Learning.

Students are instructed to access and review the preparatory materials in the morning before the online seminar. The preparation is undertaken individually. First, students read the Intercollegiate Green Theatre Checklist,^25^ an evidence-based framework promoting environmentally sustainable practices in perioperative care. The checklist provides guidance across four domains: preparing for surgery, intraoperative equipment, after the operation and anaesthesia, and highlights practical actions. For nurses, it highlights practical steps such as using reusable textiles, streamlining instrument sets, avoiding opening unnecessary supplies and sorting waste properly. The Intercollegiate Green Theatre Checklist^25^ is provided as a preparatory resource and discussion aid. As the current study involves operating theatre nursing students only, the anaesthesia-specific domain is not included in the seminar activities. Future interprofessional versions of the educational intervention may incorporate this domain to facilitate collaboration across the wider perioperative team.

Second, students review material developed by the teaching team describing operating theatre nursing education and clinical practice in Sweden and Norway. This presentation outlines key structural features of each context, including programme duration, operating theatre staffing models and selected workforce conditions, as well as differences in professional roles and responsibilities.

#### Synchronous COIL seminar (90 min)

The seminar is conducted in English and follows a common overarching structure and draws on the findings of Carter *et al*^18^ by incorporating elements of structure, collaboration and problem-oriented tasks that require analysis, justification and reflection. The seminar is facilitated by academics with expertise in perioperative nursing (MC from KI and KB from OsloMet) and observed by a senior researcher from the partner institution (AF from KI and SOD from OsloMet). The senior researcher will document fidelity, engagement and adherence to the planned format through structured field notes. The common introductory session is facilitated jointly, after which each format-specific session is facilitated by one academic.

#### Introduction and icebreaker session

The seminar begins with an introductory presentation designed to establish a shared conceptual foundation, anchor subsequent discussions within an evidence-informed framework^25^ and provide a common point of reference across both seminar formats. The introduction also foregrounds the ethical dimensions of perioperative practice by emphasising students’ professional responsibility in relation to perioperative sustainability. Students are encouraged to consider their future roles as change agents within perioperative care, highlighting the importance of critically reflecting on current practices and contributing to the development of more sustainable healthcare environments.^7^ The presentation includes an overview of the environmental impact of operating theatres and the rationale for sustainable perioperative practice. It introduces and contextualises the Intercollegiate Green Theatre Checklist^25^ and concludes with an explanation of the seminar task and intended learning outcomes. Following the presentation, students join their pre-assigned breakout rooms for an ice-breaking activity and then return to the common session. Before a scheduled break, they receive a link to the online session they should join afterwards, which is also available in the COIL course room. The link will also be posted in an announcement in the course room. Following the break, students join their assigned online session, where the format-specific component of the seminar begins.

#### Small-group discussion (40 min)

After the break, students join their assigned online session and receive the format-specific discussion instructions, after which they undertake the sustainability discussion in the same pre-assigned small groups as during the ice-breaker. Students work in small groups of 5–6 without faculty presence, in order to support open peer discussion and independent reasoning.

#### Plenary session (20 min)

Following the small-group discussions, students reconvene within their allocated learning format for a teacher-facilitated plenary session. The purpose of the plenary is to enable comparison across groups and support the articulation of reasoning related to clinical priorities, feasibility and professional responsibility. The teacher facilitates the session using structured prompts to ensure balanced participation and alignment with the intended learning objectives.

#### Format 1 (phase-specific groups with documentation in a shared digital workspace)

Each group appoints a facilitator and a timekeeper to support equal participation. The Intercollegiate Green Theatre Checklist is available as a supporting resource throughout the activity. Students will be organised around one of three perioperative phases (pre-, intra- and post-operative) and engage in an in-depth exploration of sustainability-related practices (table 2). Within their assigned phase, groups discuss current practice and document reflections and proposed actions in a shared digital workspace, guided by the prompt: ‘Within this phase, where does the operating theatre nurse genuinely have room to influence practice, and where does that influence stop?’ To minimise cross-group influence during the task, contributions from other groups remain hidden until the small-group work is completed. Students document proposed actions, as well as perceived barriers and required support, within their assigned area. Within each group, students identify and agree on the sustainability action they consider most important.

**Table 2:** Format-specific participant activities during the intervention

Phase	Format 1: phase-specific groups documenting in shared* digital board	Format 2: cross-phase groups with flexible documentation
Focus for small group interaction	Identifying and discussing sustainability-related actions within one phase (pre, intra or postoperative care phase).	Identifying and discussing sustainability-related practices across the perioperative care chain, focusing on prioritisation, feasibility and competing considerations.
Discussion prompts	Discussion is guided by a phase-specific prompt focusing on scope of influence, including where the operating theatre nurse can act and where influence is limited.	Discussion is guided by broader prompts addressing feasible actions, constraints and required support across phases.
Between-group interaction and documentation	Verbal discussion is combined with documentation in a pre-structured digital space organised by phases. Students contribute written entries reflecting actions, barriers and support needs.	Discussion is primarily verbal, and groups choose their own documentation format to capture prioritised actions, reasoning and constraints.
Plenary session	Group contributions are presented using the shared digital space, enabling comparison across phases and supporting synthesis of phase-specific and system-level insights.	Groups present summaries based on their chosen format. Plenary discussion focuses on comparing priorities, identifying convergences/divergences and making reasoning explicit across phases.

* Groups document their contributions in separate columns within the same shared digital board. Other groups’ contributions remain hidden during the small-group discussion and are made visible at the start of the plenary session.

During the plenary session, groups view the whole digital board as a visual aid to present and discuss their findings with all participants. Each group presents key outputs from their assigned phase such as identified practices, proposed actions, feasibility considerations and barriers/support needs. The teacher will invite groups to specify where influence is perceived as within or beyond the scope of the operating theatre nurse role and prompt elaboration on feasibility and constraints within the phase. The teacher can also refer to outputs to highlight patterns and variation across phases and facilitate linking of phase-specific insights to the perioperative pathway as a whole. Lastly, the teacher and students summarise key phase-specific and cross-phase findings. The facilitation focuses on clarification of scope, articulation of limits of influence and consolidation of phase-specific insights within a broader system context.

#### Format 2 (cross-phase groups with flexible documentation)

Each group appoints a facilitator and a timekeeper to support equal participation. The Intercollegiate Green Theatre Checklist is available as a supporting resource throughout the activity. Students discuss sustainability-related practices across all three phases. The discussion focuses on identifying actions that are feasible for the theatre nurses to implement as well as what challenges or constraints may arise. Students are also asked to discuss what types of support would be needed to enable implementation (table 2). The groups can choose how they document their work. Options include using a digital whiteboard, creating a presentation or appointing note-takers (one from each country where feasible) to capture key points from the discussion. Each group is required to produce a collaborative group output summarising its conclusions. Documentation should reflect prioritised issues and group reflections on feasibility and constraints.

During the plenary session, groups present their output and discuss their reasoning with all participants. Each group presents a summary of their discussion based on their chosen format (eg, digital whiteboard, PowerPoint or written notes), including proposed actions, underlying rationale and perceived barriers and enablers. The teacher will invite groups to report key priorities and associated justifications and prompt clarification of assumptions underpinning proposed actions. The teacher will also facilitate comparison across groups, including the identification of converging and diverging perspectives, and will draw attention to areas of disagreement or competing priorities across phases. Finally, the teacher and students will summarise cross-cutting themes emerging from the discussion. The facilitation focuses on making reasoning explicit and examining variation in how feasibility and responsibility are interpreted across groups.

### Outcomes

#### Primary outcomes

The primary outcomes are feasibility and acceptability of the intervention and study procedures. These include consent rate, follow-up questionnaire completion rate, completeness of questionnaire data, technical feasibility of seminar delivery and data collection procedures, intervention fidelity and adherence to the planned learning formats, participant engagement with seminar activities and the suitability of the qualitative data collection and coding framework for addressing the study aims. These outcomes will inform progression decisions for a future randomised controlled trial (table 3).

**Table 3:** Traffic-light progression criteria for a future definitive trial

Criteria	Green (proceed)	Amber (amend)	Red (major redesign)
Consent rate	≥60% of eligible students provide consent	30–59%	<30%
Follow-up survey completion	≥60% of consenting participants complete follow-up survey	30–59%	<30%
Missing questionnaire items	<10% missing data across questionnaire items	10–20%	>20%
Technical feasibility	<5% of participants report technical difficulties that prevent or impact on participation	5–10%	>10%
Qualitative data richness	Qualitative responses provide sufficient breadth and depth to address study aims	Some categories require modification or expansion	Data insufficient to address study aims
Suitability of coding framework	Existing coding framework adequately captures data with only minor refinements required	Several codes require revision or addition	Framework unsuitable and requires complete redevelopment

#### Secondary outcomes

For exploratory educational outcomes, the study will examine students’ reflective articulation of professional agency in relation to sustainable perioperative practice and perceptions of intercultural collaboration and learning. In addition, students’ attitudes towards environmentally sustainable practice will be explored quantitatively using anonymous questionnaires administered at baseline and follow-up. As responses cannot be linked at the individual level, analyses will be limited to provide preliminary descriptive information and comparisons of aggregate group responses at each time point and will not be used to assess individual-level changes on attitudes towards sustainability. The findings will inform outcome selection for a future trial.

### Data collection and tools

Data collection takes place before, during and immediately following the seminar and 1 week after (table 1).

Attitudes towards sustainability in the operating theatre will be assessed at two time points (baseline and follow-up) using a questionnaire based on ‘Attitude towards sustainability in the OT’, originally developed by Lim *et al*^26^ (online supplemental material (questionnaires)). The instrument has previously been translated and culturally adapted to the Norwegian context in a pilot study.^27^ The questionnaire comprises nine items, including one additional item adapted from a South African study,^28^ and is rated on a five-point Likert scale ranging from strongly disagree (1) to strongly agree (5). It is designed to capture a broad range of sustainability-related attitudes, including awareness of environmental impact, perceived knowledge and competence, engagement and responsibility and willingness to adopt and support sustainable practices within perioperative care. In the Norwegian validation study, the instrument demonstrated moderate internal consistency, which is considered acceptable for exploratory use. For the purpose of this study, minor adaptations will be made to ensure relevance for operating theatre nursing students nearing completion of their education.

Recruitment and participation will be monitored through enrolment records, while attrition will be assessed based on attendance and completion of study components. Engagement and adherence will be explored through attendance records and session logs with facilitator reflections, as well as through the completeness and quality of submitted student outputs. Intervention fidelity will be assessed using a structured observation protocol completed by the senior researchers (AF and SOD) who observe during the seminar. The protocol is a checklist-based assessment of adherence to the planned seminar components, documentation of protocol deviations and ratings of overall fidelity (high, moderate or low). The observation protocol is provided as online supplemental material (observation protocol).

Students’ reflective articulation of professional agency will be captured through collaborative outputs and artefacts (eg, digital boards or presentations), facilitator reflections and observer field notes. These data will form the basis for qualitative analysis.

Perceptions of intercultural collaboration and learning will be assessed using the COIL-I instrument^29^ (online supplemental material (questionnaires)). The instrument’s content validity has been established through a Delphi study assessing item clarity, coherence, relevance and sufficiency. While overall coherence and relevance were supported, some items were identified as requiring improved clarity. Accordingly, minor wording adjustments will be made to enhance comprehensibility in the present context. The original Likert response scale and response anchors of the COIL-I instrument were retained unchanged. Response options range from 1 (‘I do not agree at all’) to 6 (‘Strongly agree’), following the scoring procedures described in the original instrument. Retaining the original response format supports fidelity to the instrument and facilitates comparison with previous applications.

### Data management

All data will be handled in accordance with General Data Protection Regulation and institutional policies. Data will be stored securely on institutional servers at KI and OsloMet for up to 10 years, with access restricted to the research team. After this period, data will be deleted or archived in accordance with institutional guidelines. Survey data will be anonymous. Qualitative data will be de-identified prior to analysis. Because qualitative data consist of collaborative group outputs, individual contributions cannot be separated reliably within the educational setting. Accordingly, outputs will be treated as group-level artefacts and no attempt will be made to attribute statements to individual students. Data may be exported to standard formats (eg, Excel, Word) and analysed using statistical software (eg, SPSS).

### Data analysis

The qualitative analysis focuses on collaboratively constructed reflections and reasoning as expressed through group artefacts and observer field notes. Collaborative outputs will be treated as group artefacts and analysed at group level rather than attributed to individual students. All qualitative data sources will be analysed as part of a unified dataset. Analyses will be conducted using reflexive thematic analysis.^30 31^

The analysis will focus on identifying patterns in students’ reflective work as expressions of professional agency in relation to sustainable perioperative practice. The unit of analysis will consist of meaning units identified across these data sources. A meaning unit is defined as a segment of text, ranging from a phrase to several sentences, that expresses a coherent idea related to professional agency. Each meaning unit will be coded using a theoretically informed coding framework (online supplemental (coding scheme)). The framework integrates dimensions of agency^16^ with key sustainability competencies.^15^ The framework reflects six dimensions of professional agency: past-oriented and professional grounding, future-oriented intentions and actions, judgement in context, ethical values in sustainability reasoning, agency in interaction and collaboration and feasible transformation. Coding will be conducted iteratively, combining deductive coding based on the predefined framework with inductive refinement. Multiple codes may be applied to the same meaning unit where different dimensions of agency are expressed. Meaning units will be identified across all qualitative data sources using the same conceptual definition. The boundaries of a meaning unit will be determined by the coherence of the expressed idea rather than by the format, length or source of the text. Thus, a meaning unit may consist of a brief statement within a collaborative artefact or a longer segment within an observer field note.

The analysis is intended to explore how professional agency is expressed across different forms of student engagement. Given the theoretical focus of the study, a primarily deductive approach was chosen to support a transparent and structured analysis of students’ reflections in relation to established dimensions of professional agency and sustainability competence. Inductive analysis will complement this by identifying unanticipated aspects of students’ experiences and reasoning.

Comparative analysis will be conducted between the two study arms with a focus on patterns in how professional agency is expressed. Given the differences in documentation structure between the two formats, comparisons will not focus on the quantity of text produced or on code frequencies. Rather, the analysis will explore qualitative differences in how professional agency is expressed, articulated and integrated within students’ collaborative reasoning.

The comparison will address differences in the form of expression and the complexity of agency expressions. Complexity is defined as the extent to which multiple dimensions (eg, action, constraints, ethical reasoning and relational aspects) are integrated within the same meaning unit. Complexity will be characterised at three levels (low, moderate, high). Low complexity refers to expressions encompassing a single dimension, typically limited to proposing an action. Moderate complexity includes integration of two or more dimensions. High complexity reflects the integration of multiple dimensions, including consideration of constraints, ethical reasoning and relational aspects within the same meaning unit. Illustrative examples of each level will be developed to support analytic consistency. The comparison is intended to explore qualitatively distinct patterns in students’ collaborative reflective work, in line with the exploratory aims of the study.

To reduce the influence of facilitator preconceptions, data from each seminar will initially be coded by a researcher who did not facilitate that particular seminar. Thus, collaborative artefacts and observer field notes from the seminar facilitated by MC will be coded by KB, while data from the seminar facilitated by KB will be coded by MC. Initial coding and interpretations will subsequently be reviewed and discussed with senior researchers (AF and SOD) to explore alternative interpretations, challenge assumptions and enhance reflexive engagement with the data.

Quantitative data from questionnaires will be analysed in accordance with the scoring procedures and recommendations provided by the original instrument developers. All other quantitative data will be analysed using descriptive statistics, including medians, IQRs, proportions and other appropriate summary measures based on the level and distribution of the data. Survey data will be collected anonymously and no identifying information or participant codes will be collected. Consequently, responses from baseline and follow-up questionnaires cannot be linked at the individual level and will be analysed as unpaired aggregate datasets.

Integration will occur at the interpretation stage through a triangulation approach, in which findings from the quantitative and qualitative analyses are systematically compared. This form of integration aligns with recommendations that mixed-methods studies should explicitly examine the relationships between datasets to identify areas of convergence, divergence and complementarity.^22^ Specifically, results from the survey data on perceived learning and intercultural collaboration will be compared with qualitative themes describing students’ reflective articulation of professional agency. This comparison will allow identification of whether reported perceptions correspond with, differ from or are elaborated by the qualitative findings, thereby providing a more comprehensive understanding of the educational processes under study. Through this process, integrated interpretations (meta-inferences) will be generated, reflecting the added value of combining quantitative and qualitative data to produce a more complete understanding of how learning formats shape reflective processes and professional reasoning.^22^

### Rigour and trustworthiness

Several strategies will be used to enhance the rigour and trustworthiness of the qualitative component of the study, including triangulation of data sources, a theoretically informed coding framework, transparent documentation of the analytic process and researcher reflexivity.^32^ The inclusion of multiple qualitative data sources (collaborative outputs and field notes) enables triangulation of perspectives and supports a more comprehensive understanding of students’ expressions of professional agency. The use of a theoretically informed coding framework enhances analytical rigour by grounding interpretations in an established conceptual model of professional agency and sustainability competence. The analytic process will be documented throughout, including the development and refinement of the coding framework, to ensure transparency in how interpretations are generated. Reflexive awareness of researcher roles will be maintained throughout the study by using analytic discussions to explore alternative interpretations, challenge assumptions and refine the coding process, acknowledging the dual roles of team members in both intervention delivery and analysis. This is expected to mitigate potential confirmation bias and strengthen analytic rigour. Reflexive notes and records of analytic decisions will be maintained throughout the analysis.

### Decision-making and progression criteria

Progression to a future definitive trial will be guided by prespecified progression criteria using a traffic-light approach (green=proceed, amber=amend, red=major redesign)^33^ (table 3). Since attendance is expected as part of the educational activity and does not reflect feasibility of the research procedures, attendance is not included as a progression criterion. Criteria focus instead on recruitment, questionnaire completion, data quality, technical feasibility and the suitability of qualitative data collection and analysis. Repeated administration of the sustainability questionnaire will be considered feasible if acceptable completion rates are achieved at both measurement occasions and the collected data provide sufficient variability to assess the suitability of the instrument for future use. The assessment of qualitative data richness and the suitability of the coding framework will be undertaken after completion of data collection and preliminary qualitative analysis. These assessments will be conducted collaboratively by the qualitative research team (MC, KB, AF and SOD). Judgements will be based on whether the dataset provides sufficient breadth and depth to address the study aims and whether the predefined coding framework adequately captures the range and complexity of participants’ expressions of professional agency with only minor refinements required. Decisions will be reached through discussion and documented as part of the progression assessment process.

### Patient and public involvement

None.

## Ethics and dissemination

The study has been approved by the Norwegian Agency for Shared Services in Education and Research (reference number 266740). In Sweden, the study was reviewed by the Swedish Ethical Review Authority (reference number 2026–00252-01), which concluded that the study did not require formal ethical approval under the Swedish Ethical Review Act and issued an advisory opinion. Participation in the research component is voluntary. Eligible students will receive written Research Participant Information (online supplemental material) before each questionnaire, and submission of a questionnaire will constitute consent to participate in the research component associated with that questionnaire.

Particular care will be taken to distinguish participation in the educational activity from participation in the research and to ensure that students understand that their decision to participate or not will have no impact on their course progression, assessment or relationship with faculty. This information will be communicated clearly, both in writing and verbally, prior to consent. To protect participants’ confidentiality, survey data will be collected anonymously, and no identifying information will be linked to individual responses. Collaborative outputs will be analysed only as group artefacts, and observer field notes will focus on group processes rather than individual participants.^34^ This protocol is reported in accordance with the SPIRIT 2025 guidelines (online supplemental material).^35^

### Consent

Participation in the research component is voluntary. All students will receive written information about the study in the COIL course room. The participant information sheet (online supplemental material) will also accompany each email invitation to complete a study questionnaire. Students will be informed that participation is voluntary and that completion and submission of a questionnaire constitutes consent to participate in the research component associated with that questionnaire.

### Confidentiality

Questionnaire data will be collected anonymously, with no identifying information or participant codes linked to individual responses. Qualitative data will be de-identified and analysed at group level, with no attempt to attribute collaborative contributions to individual students.

### Data monitoring and harms

Given the educational nature of the study, no adverse events are expected. The study involves minimal risk to participants.

## Discussion

### Summary of study purpose

The study outlined in this protocol aims to assess the feasibility and acceptability of implementing and evaluating two COIL learning formats within specialist theatre nursing education. The study will generate insights into intervention delivery, study design and data collection procedures to inform a future larger-scale trial. In addition, it will exploratively examine similarities and differences in students’ reflective work and expressions of professional agency in relation to sustainable practice across the two learning formats, combining qualitative analysis with supporting quantitative data. Findings will inform decision-making regarding progression to a future trial by evaluating key uncertainties related to intervention design, data collection and analysis. These insights will guide potential refinements to the pedagogical design and determine whether to proceed to a definitive trial, with or without modifications.

### Strengths and limitations of the planned study

A key strength of the study is the use of a theoretically informed and transparent analytical framework, integrating dimensions of professional agency with sustainability competencies. This enables a structured and nuanced analysis of the students’ reflective work across multiple data sources. The inclusion of multiple qualitative and quantitative data sources, combined with a mixed-methods design, allows for a more comprehensive understanding of how different learning formats shape reflective processes. In addition, the comparative design provides a basis for examining how variations in pedagogical configuration may influence the form and complexity of the students’ expressions of professional agency.

Several limitations should be considered. The study is not designed to support statistical inference, and participation in surveys is voluntary, which may introduce response bias. Furthermore, because questionnaire responses are collected anonymously and cannot be linked across measurement occasions, the study cannot evaluate individual-level changes in students’ attitudes towards sustainability. Any differences between baseline and follow-up responses should therefore be interpreted descriptively at the aggregate level. The analysis focuses on articulated reflections rather than observed behavioural change, limiting conclusions about how learning translates into clinical practice. Moreover, the two intervention formats should be understood as distinct educational packages rather than as a controlled comparison isolating the effect of phase-specific task allocation alone. Although both formats share core components, including international small-group collaboration, facilitated discussion, a requirement to identify a priority sustainability action and plenary presentation, the formats differ in how collaborative work is organised and synthesised. Consequently, any observed differences between study arms should be interpreted as reflecting the combined influence of these pedagogical features rather than the isolated effect of task allocation across perioperative phases.

The study may also be subject to cross-contamination between groups, as the students are enrolled in the same educational context and may share experiences across study arms. Furthermore, blinding is not feasible due to the nature of the educational intervention, which may influence the participants’ responses. Finally, the study is conducted within perioperative nursing programmes in a Scandinavian context with specific organisational and educational conditions, which may affect the transferability of the findings.

### Pedagogical contribution

The study contributes pedagogically by comparing two COIL formats designed to support different forms of reflective work. The phase-specific format emphasises depth, role clarity and recognition of bounded influence within perioperative phases, whereas the cross-phase format foregrounds negotiation, prioritisation and relational dimensions of professional influence. In line with sustainability education frameworks,^15^ the activity promotes reflective dialogue and the integration of multiple perspectives to support context-sensitive decision-making in sustainable practice.

### Transferability to nursing curricula

Although situated within theatre nursing education, the underlying pedagogical principles, structured reflection on clinical experience, international peer dialogue and an explicit focus on professional agency are transferable across nursing contexts and educational levels. The COIL formats are feasible within existing curricula, relying on short, synchronous online sessions without requiring physical mobility, making them particularly relevant in settings prioritising internationalisation and sustainability.

### Implications for future research

The study will provide insight into the suitability of the pedagogical design, data collection methods and analytical framework for capturing students’ expressions of professional agency. It may also identify preliminary differences in how agency is expressed across learning formats, informing refinement of the intervention and outcome measures. These findings will guide the design of a future trial exploring the development of professional agency over time and its potential links to clinical practice.

## Supplementary material

10.1136/bmjopen-2026-124998 online supplemental file 1

10.1136/bmjopen-2026-124998 online supplemental file 2

10.1136/bmjopen-2026-124998 online supplemental file 3

10.1136/bmjopen-2026-124998 online supplemental file 4

10.1136/bmjopen-2026-124998 online supplemental file 5

## Data Availability

Data sharing not applicable as no datasets generated and/or analysed for this study. Data are available upon reasonable request.
