# Supplementary material for: Evaluating learning formats in international collaborative nursing education on professional agency in perioperative sustainability: protocol for a mixed-methods, randomised pilot study
Source: BMJ Open. 2026 Nov 11;16(11):e124998. doi: 10.1136/bmjopen-2026-124998 (PMC13756281; doi:10.1136/bmjopen-2026-124998)
Supplement: Supplementary file 1 [file bmjopen-16-11-s001.pdf]

Observer: ______________________
Seminar format:
☐ Format 1: Phase-specific groups with collaborative digital documentation
☐ Format 2: Cross-phase groups with flexible documentation
Number of participants:______

Intervention Fidelity

Indicate whether the following elements were delivered as planned:

Introduction (15 min)

☐ Introduction to sustainable perioperative practice

☐ Overview of environmental impacts associated with operating theatres

☐ Introduction to the Intercollegiate Green Theatre Checklist

☐ Explanation of seminar task and learning objectives

Comments:____

Small-group discussions (40 min)

☐ Groups consisted of approximately 5-6 students

☐ Balanced group composition achieved

☐ Facilitator appointed within group

☐ Timekeeper appointed within group

☐ Documentation completed according to assigned format

☐ Group task appeared to be understood

Comments:____

Plenary session (20 min)

☐ All groups presented their work

☐ Discussion of feasibility and barriers occurred

☐ Comparison across groups was facilitated

☐ Sustainability actions were discussed

☐ Session concluded with a summary of key points

Comments:____

How closely was the intervention delivered according to protocol?

☐ High fidelity

☐ Moderate fidelity

☐ Low fidelity

Rationale:____

Participant Engagement

**Aspect**	**Comments**	**Low**	**Moderate**	**High**
Active participation in discussions		☐	☐	☐
Equal participation within groups		☐	☐	☐

Interaction between Swedish and Norwegian students		☐	☐	☐
Engagement during plenary discussion		☐	☐	☐
Overall engagement with seminar task		☐	☐	☐

Reflective Work and Expressions of Professional Agency

Note illustrative examples, quotations, or discussion points observed during the seminar.

Past-oriented and professional grounding

Students referred to:

- Previous clinical experiences
- Existing routines or practices
- Professional norms or expectations

Examples:____

Future-oriented intentions and actions

Students proposed:

- New initiatives
- Improvements to practice
- Future sustainability actions

Examples:____

Judgement in context (practical-evaluative reasoning)

Students discussed:

- Feasibility
- Prioritisation
- Organisational constraints
- Available resources

Examples:____

Ethical reasoning and values

Students reflected on:

- Patient safety
- Environmental considerations
- Resource use
- Competing priorities

Examples:____

Agency in interaction and collaboration

Students:

- Built on each other's ideas
- Negotiated shared positions
- Compared Swedish and Norwegian perspectives
- Engaged in collaborative reasoning

Examples:____

Feasible transformation

Students identified:

- Concrete actions that could realistically be implemented
- Necessary support structures
- Organisational conditions required for change

Examples:____

Format-Specific Observations

Format 1: Phase-Specific Groups

Comment on:

- Extent to which discussion remained focused on the assigned perioperative phase
- Discussion regarding the operating theatre nurse's scope of influence
- Use of the digital workspace
- Documentation of actions, barriers, and support needs

Comments:____

Format 2: Cross-Phase Groups

Comment on:

- Integration of multiple perioperative phases
- Prioritisation among competing sustainability actions
- Negotiation of different perspectives
- Usefulness and quality of chosen documentation format

Comments:____

Observer Reflections

What aspects of the seminar appeared to support students' reflection on sustainability and professional agency?

Were there any unexpected events, challenges, or notable observations that should be considered when planning a future trial?

Additional field notes
