# Supplementary material for: Evaluating learning formats in international collaborative nursing education on professional agency in perioperative sustainability: protocol for a mixed-methods, randomised pilot study
Source: BMJ Open. 2026 Nov 11;16(11):e124998. doi: 10.1136/bmjopen-2026-124998 (PMC13756281; doi:10.1136/bmjopen-2026-124998)
Supplement: Supplementary file 2 [file bmjopen-16-11-s002.pdf]

International Digital Seminars on Sustainable Operating Theatre Nursing Care – Reflection and Dialogue among Specialist Nursing Students Pilot Study using COIL (Collaborative Online International Learning) between Karolinska Institutet and OsloMet

Information for Participants
We would like to invite you to take part in a research project. This document explains what the project is about and what participation involves.

What is this project, and why are we asking you to take part?
We want to explore how a digital seminar with students from different countries can support future perioperative nurses in learning about sustainability and sharing experiences.
You are being invited because you are enrolled in the specialist nursing programme in perioperative care.

The project's responsible institution ("research principal") is Karolinska Institutet and OsloMet. This means that KI and OsloMet are accountable for the study.

What will you be asked to do?
You will participate in a 90-minute online seminar in English together with students from OsloMet and KI. If you agree to participate in the study, you will be asked to complete three short online questionnaires: one before the seminar, one immediately after the seminar, and one approximately one week later. The questionnaires ask about your attitudes towards sustainability in the operating theatre and your experience of intercultural collaboration and learning during the seminar. Each questionnaire takes approximately 5 minutes to complete.

The seminar is part of your regular course, but participation in the research study is voluntary. Choosing not to participate in the study will not affect your studies, grades, or participation in the seminar.

Possible consequences and risks
There are no known risks associated with participating.
Some students may feel a bit unsure about speaking English, but your programme already uses English-language course literature, and you will receive support during the seminar.

What happens to your data?
The project will collect and store some information about you.
We will collect:
Your survey responses (text and multiple-choice answers)

How your data will be handled:
All data will be de-identified before analysis – your name or personal identity number will not be included in the analysis file.
No audio or video recordings will be made.
Data from Sweden will be stored on secure KI servers (KI Survey/text files).
Only the project team will have access to the data.

Purpose
To understand how students experience the seminar and what sustainability-related ideas emerge.

Your consent to participate in the research study.
Your data will be handled so that no unauthorised person can access it.
Karolinska Institutet is responsible for the processing of your personal data.
Under the EU General Data Protection Regulation (GDPR), you have the right to access your data, request corrections, and - when possible - request deletion or limitation of data processing. However, the right to deletion or limitation does not apply if the data is necessary for this research project.

If you want to access your data, please contact:
Marie-Therese Crafoord, marie-therese.crafoord@ki.se, +46 708 732 285

The Data Protection Officer at KI can be reached at dataskyddsombud@ki.se.
If you are not satisfied with how your data is handled, you may file a complaint with the Swedish Authority for Privacy Protection (Integritetsskyddsmyndigheten).
No biological samples are collected in this project.

How will you receive information about the results?
The results of the project will be published as a scientific article.
Individual survey responses or chat comments will not be reported back to participants.

Insurance and compensation
Students are covered by Kammarkollegiet's personal injury insurance.
You will not receive financial compensation for participation.
(The project does not involve any physical procedures.)

Voluntary participation
Your participation is voluntary. You may withdraw at any time without giving a reason.
Declining or withdrawing will not affect your future teaching or assessment in any way.
If you wish to withdraw, please contact the project leader (see below).

Project contacts
Marie-Therese Crafoord
+46 8-524 867 67
marie-therese.crafoord@ki.se
Karianne Barlein
+47 915 87 529
kakar@oslomet.no

Consent to participate
You consent to participating in the study by replying to the questionnaire.
