# Supplementary material for: Evaluating learning formats in international collaborative nursing education on professional agency in perioperative sustainability: protocol for a mixed-methods, randomised pilot study
Source: BMJ Open. 2026 Nov 11;16(11):e124998. doi: 10.1136/bmjopen-2026-124998 (PMC13756281; doi:10.1136/bmjopen-2026-124998)
Supplement: Supplementary file 3 [file bmjopen-16-11-s003.pdf]

HT26 Perioperative Sustainability Baseline

Time required: About 5–7 minutes

Instructions: Please answer the following questions based on your experiences during your clinical placement in the operating theatre department. There are no right or wrong answers. We are interested in your perceptions and experiences regarding environmental sustainability and sustainable practices in the operating theatre.

For each statement, indicate the extent to which you agree or disagree.

This questionnaire is based on the instrument described by Erstad (2025).

Erstad AS. *Operasjonssykepleieres holdninger til miljøvennlige tiltak og bærekraftig praksis i operasjonsavdelingen: oversettelse, validering og testing av "Attitude towards sustainability in the OT" på operasjonssykepleiere i norsk kontekst* [master's thesis]. Oslo: OsloMet – Storbyuniversitetet; 2025.

Q1

1. I am aware of the environmental impact of the operating theatre department.

- Strongly disagree
- Disagree
- Neither agree nor disagree
- Strongly agree

Q5

2. I feel that I have sufficient knowledge about the environmental impact of the operating theatre
department to make environmentally sustainable decisions.

- Strongly disagree
- Disagree
- Neither agree nor disagree
- Strongly agree

Q2

3. I am confident in my knowledge of how to sort waste correctly according to operating theatre
department's waste management routines.

- Strongly disagree
- Disagree
- Neither agree nor disagree
- Strongly agree

Q3

4. I care about how the work carried out in operating theatre departments affects the environment
and sustainability.

- Strongly disagree
- Disagree
- Neither agree nor disagree
- Strongly agree

Q4

5. I practice, or strive to implement, sustainable behaviours in my daily life outside the operating
theatre department.

- Strongly disagree
- Disagree
- Neither agree nor disagree
- Strongly agree

Q6

6. I am willing to change my own practice in the operating theatre department to reduce environmental impact.

- Strongly disagree
- Disagree
- Neither agree nor disagree
- Strongly agree

Q7

7. Operating theatre departments implements sufficient sustainability measures.

- Strongly disagree
- Disagree
- Neither agree nor disagree
- Strongly agree

Q8

8. Operating theatre department should adopt more sustainable practices.

- Strongly disagree
- Disagree
- Neither agree nor disagree
- Strongly agree

Q9

9. To increase recycling and recovery of all waste generated in the operating theatre department, I
am willing to contribute by spending time learning about sustainable waste management practices
myself.

- ◯ Strongly disagree
- ◯ Disagree
- ◯ Neither agree nor disagree
- ◯ Strongly agree

Q10

10. . To increase recycling and recovery of all waste generated in the operating theatre department, I am willing to contribute by spending time helping others learn about sustainable waste management
practices.

- ◯ Strongly disagree
- ◯ Disagree
- ◯ Neither agree nor disagree
- ◯ Strongly agree

HT26 COIL Learning Evaluation

Time required: About 7-10 minutes

Instructions: Answer based on your experience of today's seminar. There are no right or wrong answers. For the open-text questions, you may answer in English, Swedish, or Norwegian.

The following items are adapted from the COIL scale developed by Rozo-García, Alcantar-Nieblas and Ramírez-Montoya (2024). The original scale is published under a Creative Commons Attribution (CC BY) License. Rozo-García H, Alcantar-Nieblas C, Ramírez-Montoya MS. Scale to measure student perception in collaborative online international learning experiences: design and validation. Front Educ. 2024;9:1401295. doi:10.3389/feduc.2024.1401295.

Q10

1. Which topic did you find most interesting and meaningful?

- ◯ Resource use
- ◯ Waste and waste handling
- ◯ Priorities and ethical choices

Q11

2. How relevant did you find the discussion on sustainable intraoperative practices for your future professional role?

- ◯ Not relevant at all
- ◯ A little relevant
- ◯ Mostly relevant

Q12

3. To what degree did the seminar increase your understanding of sustainability aspects in perioperative care?

- ◯ Not at all
- ◯ A little
- ◯ Mostly

Q13

4. How well did the seminar allow for critical reflection on sustainability issues?

- ◯ Not at all
- ◯ A little
- ◯ Mostly

Q14

5. How meaningful was it to compare the checklist’s recommendations with your own experiences?

- ◯ Not meaningful at all
- ◯ A little meaningful
- ◯ Mostly meaningful

Q16

6. What worked well with the technology, and what could be improved?

(Open-text answer)

Q17

7. It was easy to learn with my peers through interaction in digital environments.

- ◯ I do not agree at all
- ◯ I do not agree
- ◯ Somewhat disagree
- ◯ Neutral
- ◯ Somewhat agree
- ◯ Strongly agree

Q1

8. I could communicate adequately with peers from other countries through tools in digital environments.

- ◯ I do not agree at all
- ◯ I do not agree
- ◯ Somewhat disagree
- ◯ Neutral
- ◯ Somewhat agree
- ◯ Strongly agree

Q18

9. I respected the views of my peers in the online course, even if I disagreed with them.

- ◯ I do not agree at all
- ◯ I do not agree
- ◯ Somewhat disagree
- ◯ Neutral
- ◯ Somewhat agree
- ◯ Strongly agree

Q19

10. My peers considered my opinions, even if they differed from theirs.

- I do not agree at all
- I do not agree
- Somewhat disagree
- Neutral
- Somewhat agree
- Strongly agree

Q20

11. Interaction with my peers in digital environments favoured my learning.

- I do not agree at all
- I do not agree
- Somewhat disagree
- Neutral
- Somewhat agree
- Strongly agree

Q21

12. The teacher of the online seminar favoured that we had a collaborative environment among
peers.

- I do not agree at all
- I do not agree
- Somewhat disagree
- Neutral
- Somewhat agree
- Strongly agree

Q22

13. I perceived a cooperative environment among most peers during the online seminar.

- I do not agree at all
- I do not agree
- Somewhat disagree
- Neutral
- Somewhat agree
- Strongly agree

Q23

14. My online seminar peers and I were able to guide each other to meet the objectives despite
language barriers.

- I do not agree at all
- I do not agree
- Somewhat disagree
- Neutral
- Somewhat agree
- Strongly agree

Q24

15. The collaborative work with my peers helped me understand different ways of organising work and tasks.

- I do not agree at all
- I do not agree
- Somewhat disagree
- Neutral
- Somewhat agree
- Strongly agree

Q25

16. Working collaboratively with peers from another culture helped me develop my practical
communication skills.

- I do not agree at all
- I do not agree
- Somewhat disagree
- Neutral
- Somewhat agree
- Strongly agree

Q26

17. Working collaboratively with peers from another culture helped me to develop my leadership
skills.

- I do not agree at all
- I do not agree
- Somewhat disagree
- Neutral
- Somewhat agree
- Strongly agree

Q27

18. Collaborative work with peers from another culture made me commit to my tasks.

- I do not agree at all
- I do not agree
- Somewhat disagree
- Neutral
- Somewhat agree
- Strongly agree

Q28

19. I felt satisfied that I successfully achieved the seminar objectives.

- I do not agree at all
- I do not agree
- Somewhat disagree
- Neutral
- Somewhat agree
- Strongly agree

Q29

20. Working with peers from a different culture helped me remember that everyone is different and must be considered when working in a group.

- I do not agree at all
- I do not agree
- Somewhat disagree
- Neutral
- Somewhat agree
- Strongly agree

Q30

21. Working with peers from a different culture helped me learn about new digital tools.

- I do not agree at all
- I do not agree
- Somewhat disagree
- Neutral
- Somewhat agree
- Strongly agree

Q31

22. Working with peers from a different culture helped me realize how important respect is.

- I do not agree at all
- I do not agree
- Somewhat disagree
- Neutral
- Somewhat agree
- Strongly agree

Q32

23. Working with peers from a different culture helped me realize that we are not so different.

- I do not agree at all
- I do not agree
- Somewhat disagree
- Neutral
- Somewhat agree
- Strongly agree

Q33

24. Working cross-culturally helped me to reflect on global issues.

- I do not agree at all
- I do not agree
- Somewhat disagree
- Neutral
- Somewhat agree
- Strongly agree

Q34

25. Working with people from a different culture helped me to learn about study habits that can help me in the future.

- I do not agree at all
- I do not agree
- Somewhat disagree
- Neutral
- Somewhat agree
- Strongly agree

Q2

26. Did you prepare for the seminar by reading the material sent in advance?

- Yes
- No

Q3

27. If you answered “Yes” What did you read, and how did you prepare? (open-text)

Q6

28. Which language(s) did you use most when participating in the discussion?

(Check the one that fits best.)

- English
- Norwegian
- Swedish
- Mixed (Swedish/Norwegian and English)

Q7

29. How did the language affect your ability to participate and learn during the seminar?

- Made it much harder
- Made it somewhat harder
- Neither nor
- Made it somewhat easier
- Made it much easier

Q8

30. What could have made the language use even more supportive for your learning?

(Open-text answer)

HT26 Perioperative Sustainability Follow-up

Time required: About 5–7 minutes

Instructions: Please answer the following questions based on your experiences during your clinical placement in the operating theatre department. There are no right or wrong answers. We are interested in your perceptions and experiences regarding environmental sustainability and sustainable practices in the operating theatre.

For each statement, indicate the extent to which you agree or disagree.

This questionnaire is based on the instrument described by Erstad (2025).

Erstad AS. *Operasjonssykepleieres holdninger til miljøvennlige tiltak og bærekraftig praksis i operasjonsavdelingen: oversettelse, validering og testing av "Attitude towards sustainability in the OT" på operasjonssykepleiere i norsk kontekst* [master's thesis]. Oslo: OsloMet – Storbyuniversitetet; 2025.

Q1

1. I am aware of the environmental impact of the operating theatre department.

- Strongly disagree
- Disagree
- Neither agree nor disagree
- Strongly agree

Q2

2. I feel that I have sufficient knowledge about the environmental impact of the operating theatre department to make environmentally sustainable decisions.

- Strongly disagree
- Disagree
- Neither agree nor disagree
- Strongly agree

Q3

3. I am confident in my knowledge of how to sort waste correctly according to operating theatre department's waste management routines.

- Strongly disagree
- Disagree
- Neither agree nor disagree
- Strongly agree

Q4

4. I care about how the work carried out in operating theatre departments affects the environment
and sustainability.

- Strongly disagree
- Disagree
- Neither agree nor disagree
- Strongly agree

Q5

5. I practice, or strive to implement, sustainable behaviours in my daily life outside the operating
theatre department.

- Strongly disagree
- Disagree
- Neither agree nor disagree
- Strongly agree

Q6

6. I am willing to change my own practice in the operating theatre department to reduce environmental impact.

- Strongly disagree
- Disagree
- Neither agree nor disagree
- Strongly agree

Q7

7. Operating theatre departments implements sufficient sustainability measures.

- Strongly disagree
- Disagree
- Neither agree nor disagree
- Strongly agree

Q8

8. Operating theatre department should adopt more sustainable practices.

- Strongly disagree
- Disagree
- Neither agree nor disagree
- Strongly agree

Q9

9. To increase recycling and recovery of all waste generated in the operating theatre department, I
am willing to contribute by spending time learning about sustainable waste management practices
myself.

- Strongly disagree
- Disagree
- Neither agree nor disagree
- Strongly agree

Q10

10. . To increase recycling and recovery of all waste generated in the operating theatre department, I am willing to contribute by spending time helping others learn about sustainable waste management
practices.

- Strongly disagree
- Disagree
- Neither agree nor disagree
- Strongly agree
