# Supplementary material for: Evaluating learning formats in international collaborative nursing education on professional agency in perioperative sustainability: protocol for a mixed-methods, randomised pilot study
Source: BMJ Open. 2026 Nov 11;16(11):e124998. doi: 10.1136/bmjopen-2026-124998 (PMC13756281; doi:10.1136/bmjopen-2026-124998)
Supplement: Supplementary file 4 [file bmjopen-2026-124998.reviewer_comments.pdf]

PEER REVIEW HISTORY

BMJ Open publishes all reviews undertaken for accepted manuscripts. Reviewers are asked to complete a checklist review form and are provided with free text boxes to elaborate on their assessment. These free text comments are reproduced below.

ARTICLE DETAILS

Title (Provisional)

Evaluating learning formats in international collaborative nursing education on professional agency in perioperative sustainability: protocol for a mixed-methods, randomised pilot study

Authors

Crafoord, Marie-therese; Barlein , Karianne; Torbjörnsson, Eva; Vikan, Magnhild; Macintosh Kvalvik, Ida Andrea; Fagerdahl, Ami; Danielsen, Stein Ove

---

VERSION 1 - REVIEW

**Reviewer**	**1**
**Name**	**Berger-Estilita, Joana**
**Affiliation**	**Inselspital University Hospital Bern, Department of Anaesthesiology and Pain Medicine**
**Date**	**27-Aug-2026**
**COI**	**None**

General Impression:

This study protocol tackles a crucial and timely topic: embedding environmental sustainability into specialised operating theatre nursing education through Collaborative Online International Learning (COIL). The focus on professional agency rather than passive knowledge acquisition is a distinct strength, as is the attempt to test two active pedagogical formats rather than relying on a passive no-intervention comparator. The mixed-methods approach is well aligned with the exploratory nature of a feasibility pilot.

However, several aspects of the methodology, ethical execution, and analytical strategy require substantial clarification and refinement before the protocol is ready for publication.

Major Points:

- Pre-pilot Data in a Protocol Manuscript

The inclusion of pilot data and narrative evaluation (including Table 3 and statements that the pre-pilot "supported feasibility and acceptability") is unusual in a study protocol.

Methodological protocol guidelines generally require that study findings and conclusions are not reported prior to completion of the full study.

Please condense the pre-pilot description into a brief paragraph in the background/methods section to explain how prior work informed the current design, and remove Table 3 and any evaluative conclusions. If these data must remain, clearly justify why a completed four-student exercise belongs in a prospective protocol.

- Allocation Concealment and Randomisation Logistics

The protocol mentions that an educator/researcher who facilitates one of the arms will also generate the randomisation sequence. This creates a clear risk of selection and facilitator bias.

Please delegate sequence generation and participant allocation to an independent researcher not involved in teaching, facilitating breakout rooms, or assessing outcomes. Clarify precisely when and how allocations are revealed, and how access to the platform modules is restricted so neither students nor facilitators are unblinded prematurely.

- Cluster Effects and Unit of Allocation

While individual students are randomised, the learning activities and qualitative artefacts are generated within small collaborative groups.

Explicitly state whether the individual or the group is the unit of allocation and analysis. Clarify the target group size, how many groups will run per arm, and how national representation (Swedish vs. Slovenian students) will be balanced across groups. Also, clarify the discrepancy regarding sample size: the text notes a total cohort of 24–36, yet describes plenary sessions containing "approximately 20" students.

- Reconciling Anonymous Surveys with Pre–Post Tracking

The text states that survey responses will be entirely anonymous with no identifiers collected, but simultaneously plans a pre- and post-intervention assessment (baseline vs. 1-week follow-up).

True pre–post evaluation requires linked data. Clarify whether you intend to use pseudo-anonymous participant codes (e.g., self-generated alphanumeric IDs) to pair records, or if data will merely be analysed as cross-sectional, unpaired aggregates. If unpaired, avoid referring to it as within-person change.

- Timing Inconsistency in the Sustainability Instrument (Supplementary File 1)

Item Q1 ("Before participating in the seminar, I was aware of the environmental impact...") is written in the past tense. Administering this exact item at baseline does not make sense chronologically because the seminar has not occurred yet.

Rephrase baseline items into the present tense (e.g., "I am aware of...") or separate the pre-test awareness check from a post-hoc retrospective reflection.

- Feasibility Progression Criteria Need Explicit Benchmarks

The current progression criteria rely heavily on subjective terms (e.g., whether students "engage in all components" or provide "rich expressions").

Define clear, numeric Stop/Amend/Go thresholds where feasible (e.g., target consent rate ≥75%, attendance rate ≥85%, survey completion ≥80%, and maximum allowable technical dropouts). Pre-specifying these metrics is critical for determining whether a full-scale trial is justified.

- Consent Boundaries in Mandatory Collaborative Environments

The educational seminar is compulsory, but participation in the study is voluntary. In a shared collaborative workspace or plenary chat, consenting and non-consenting students will inevitably work together.

Could you detail how you will isolate or redact qualitative contributions from students who decline consent, particularly within shared Miro boards, chat transcripts, and observer field notes? Ensure students understand how their coursework is partitioned from research data without academic penalty.

- Qualitative and Mixed-Methods Analytical Depth

The coding framework based on Jääskelä et al. is sound, but operational details are sparse:

How will unitisation work across diverse data types (individual reflection texts vs. collaborative Miro notes vs. chat logs)?

How will you avoid bias given that Format A uses a structured template while Format B uses an open workspace (which inherently alters the volume and density of written text)?

Describe how the collaborative coding process will work in practice without forcing rigid inter-rater reliability onto what is framed as a reflexive thematic analysis.

- Confounding Variables Between Study Arms

Formats A and B differ across several variables at once: single-phase vs. cross-phase tasks, structured vs. unstructured boards, and isolated vs. shared plenary synthesis.

Please acknowledge in the discussion that the study evaluates two distinct "pedagogical packages" rather than isolating the single effect of phase-specific task allocation.

- Intervention Fidelity Protocols

Fidelity is stated as a key feasibility metric, yet the observation guides and adherence monitoring processes are barely described. Outline whether observers will use a structured checklist, what constitutes a protocol deviation during seminar delivery, and whether facilitators receive a standardized briefing/manual.

Minor Points:

Terminology: Standardise the terminology used for the intervention arms across the text, tables, and appendices (currently alternating between "phase-based," "phase-structured," "cross-phase," and "domain-based").

Green Theatre Checklist Alignment: Clarify the mapping between the four checklist domains (which include anaesthesia) and the three perioperative phases (pre-, intra-, post-op) assigned to the nursing students.

COIL-I Response Scale: Verify the Likert scale phrasing in the adapted COIL-I, ensuring balanced positive/negative anchors across the options.

Grammar & Mechanics: Correct minor phrasing issues throughout the manuscript (e.g., "Evidence indicate"

→ "Evidence indicates"; "departments implements" → "departments implement"; ensure proper possessive apostrophes in "participants' responses").

Study Dates: Insert the projected calendar dates for recruitment, seminar delivery, and follow-up data collection to meet standard reporting requirements.

VERSION 1 - AUTHOR RESPONSE

**Reviewer 1**	
**Major points**	
Pre-pilot Data in a Protocol Manuscript, The inclusion of pilot data and narrative evaluation (including Table 3 and statements that the pre-pilot "supported feasibility and acceptability") is unusual in a study protocol. Methodological protocol guidelines generally require that study findings and conclusions are not reported prior to completion of the full study.<br>Please condense the pre-pilot description into a brief paragraph in the background/methods section to explain how prior work informed the current design, and remove Table 3 and any evaluative conclusions. If these data must remain, clearly justify why a completed four-student exercise belongs in a prospective protocol.	Thank you for this important comment. We agree that the previous version gave undue emphasis to the pre-pilot findings and could be interpreted as reporting study results within a protocol manuscript. We have therefore substantially revised this section.<br><br>• The description of the pre-pilot has been condensed and relocated to the Methods section, immediately before the description of the educational intervention.<br>• Table 3 has been removed, together with all quantitative data and evaluative statements regarding feasibility and acceptability.<br>• We now describe the pre-pilot solely to explain how feedback from four operating theatre nursing students from the two participating institutions informed refinements of the intervention, including preparatory materials, instructions, timing, and facilitation procedures.<br>• We have clarified that the pre-pilot formed part of the development phase of the intervention and include it to provide transparency regarding the intervention development process.

Allocation Concealment and Randomisation Logistics - The protocol mentions that an educator/researcher who facilitates one of the arms will also generate the randomisation sequence. This creates a clear risk of selection and facilitator bias.<br>Please delegate sequence generation and participant allocation to an independent researcher not involved in teaching, facilitating breakout rooms, or assessing outcomes. Clarify precisely when and how allocations are revealed, and how access to the platform modules is restricted so neither students nor facilitators are unblinded prematurely.	Thank you for this important comment. We agree that the original description of the allocation procedures required further clarification. We have revised the manuscript to strengthen allocation concealment and reduce the potential risk of selection and facilitator bias.<br>• The randomisation sequence will now be generated by a member of the research team who is not involved in teaching, intervention delivery, outcome assessment, or data collection.<br>• We have also clarified the timing and procedure for allocation disclosure, specifying that students remain unaware of their allocation until the day of the intervention, when access to their allocated seminar module is released through the COIL course room.<br>• In addition, the manuscript now explains that participants only have access to materials and seminar links associated with their allocated study arm and are not provided with information about the alternative format. Although participants know which seminar they attend, they are not informed of the specific pedagogical differences between the two study arms, as the format-specific discussion prompts are introduced only during the seminar immediately before the small-group discussions.<br><br>Revisions have been made in the sections on Randomisation and allocation and Blinding.
Reconciling Anonymous Surveys with Pre–Post Tracking - The text states that survey responses will be entirely anonymous with no identifiers collected, but simultaneously plans a pre- and post-intervention assessment (baseline vs. 1-week follow-up).<br>True pre–post evaluation requires linked data. Clarify whether you intend to use pseudo-anonymous participant codes (e.g., self-generated alphanumeric IDs) to pair records, or if data will merely be analysed as cross-sectional, unpaired aggregates. If unpaired, avoid referring to it as within-person change.	Thank you for identifying this inconsistency. The intention of this pilot study is to retain full respondent anonymity and therefore no participant identifiers or self-generated codes will be collected. Consequently, baseline and follow-up survey responses will not be linked at the individual level and will be analysed as unpaired aggregate datasets. We have revised the manuscript to clarify this throughout the sections on aims, outcomes, data management and statistical analysis. References suggesting within-person change have been removed. The primary purpose of the repeated survey administration in this pilot is to assess feasibility and generate descriptive information to inform the design of a future trial, where linked individual-level data may be considered.
Timing Inconsistency in the Sustainability Instrument (Supplementary File 1) - Item Q1 ("Before participating in the seminar, I was aware of the environmental impact...") is written in the past tense. Administering this exact item at baseline does not make sense chronologically because the seminar has not occurred yet. Rephrase baseline items into the present tense (e.g., "I am aware of...") or separate the pre-test awareness check from a post-hoc retrospective reflection.	Thank you for this valuable comment. We have revised Item Q1 to ensure chronological consistency. The baseline version now uses present tense ("I am aware of the environmental impact of the operating theatre department"), while the follow-up version retains the retrospective wording ("Before participating in the seminar, I was aware of the environmental impact of the operating theatre department").

Feasibility Progression Criteria Need Explicit Benchmarks - The current progression criteria rely heavily on subjective terms (e.g., whether students "engage in all components" or provide "rich expressions").<br>Define clear, numeric Stop/Amend/Go thresholds where feasible (e.g., target consent rate ≥75%, attendance rate ≥85%, survey completion ≥80%, and maximum allowable technical dropouts). Pre-specifying these metrics is critical for determining whether a full-scale trial is justified.	Thank you for this important comment. We agree that progression criteria should be defined more explicitly. The manuscript has therefore been revised to include prespecified stop-amend-go criteria using a traffic-light approach. As this is an educational intervention delivered within an existing course, attendance is not considered a progression criterion. Instead, progression will be evaluated based on consent rates, follow-up questionnaire completion, missing data, technical feasibility, and the adequacy of the qualitative data and coding framework. We selected pragmatic thresholds appropriate for a pilot feasibility study, where the primary aim is to assess study procedures and identify areas requiring refinement before a future definitive trial.
Consent Boundaries in Mandatory Collaborative Environments - The educational seminar is compulsory, but participation in the study is voluntary. In a shared collaborative workspace or plenary chat, consenting and non-consenting students will inevitably work together.<br>Could you detail how you will isolate or redact qualitative contributions from students who decline consent, particularly within shared Miro boards, chat transcripts, and observer field notes? Ensure students understand how their coursework is partitioned from research data without academic penalty.	Thank you for this important observation. We agree that voluntary research participation within a mandatory educational activity requires particular attention to consent procedures and the distinction between educational participation and research data collection.<br>To address this, we have clarified that all students will participate fully in the educational seminar as part of the curriculum, regardless of research participation. Consent for the research component will be indicated through voluntary completion and submission of the study questionnaires. Students who choose not to participate in the research will not be required to complete any questionnaires and this decision will have no academic consequences.<br>We have revised the data collection procedures. Individual chat-based reflections will no longer be collected as research data. Collaborative outputs (such as Padlets) will be analysed as group artefacts rather than as individual contributions, and no attempt will be made to attribute specific content to named or identifiable participants. Similarly, observer field notes will focus on group-level processes, interaction patterns, and implementation characteristics rather than individual participants. These procedures are consistent with ethical guidance for educational research emphasising voluntary participation, minimisation of coercion, and protection of students who decline research participation.<br>Relevant revisions have been made to the sections on data collection, data management, confidentiality, and ethics.
Qualitative and Mixed-Methods Analytical Depth - The coding framework based on Jääskelä et al. is sound, but operational details are sparse:<br>How will unitisation work across diverse data types (individual reflection texts vs. collaborative Miro notes vs. chat logs)?	We have clarified that meaning units will be identified using the same conceptual definition across data sources, with unit boundaries determined by the coherence of the expressed idea rather than the format or length of the text.

How will you avoid bias given that Format A uses a structured template while Format B uses an open workspace (which inherently alters the volume and density of written text)?	Thank you for this important observation. We agree that given the differences in documentation structure between the two formats, comparisons should not focus on the quantity of text produced or on code frequencies alone. Rather, the analysis will explore qualitative differences in how professional agency is expressed, articulated, and integrated within students' collaborative reasoning. We have now revised the manuscript accordingly.
Describe how the collaborative coding process will work in practice without forcing rigid inter-rater reliability onto what is framed as a reflexive thematic analysis.	Thank you for this valuable suggestion. We agree that the original manuscript did not sufficiently describe the practical conduct of the collaborative coding process. We have revised the Data Analysis section to provide greater transparency regarding the analytic procedures. Specifically, initial coding will be conducted by a researcher who did not facilitate the respective seminar. Thus, collaborative artefacts and observer field notes from the seminar facilitated by MC will be coded by KB, while data from the seminar facilitated by KB will be coded by MC. This approach was chosen to reduce the influence of facilitator preconceptions during the initial stages of analysis.<br>Following initial coding, interpretations will be reviewed and discussed with senior researchers (AF and SOD). These discussions will be used to explore alternative interpretations, challenge assumptions, and support reflexive engagement with the data. Consistent with a reflexive thematic analysis approach, the purpose of collaborative discussions is not to establish statistical agreement or inter-rater reliability, but rather to enhance analytic depth, transparency, and reflexivity. Analytic decisions and reflections will be documented throughout the analysis process.
Confounding Variables Between Study Arms - Formats A and B differ across several variables at once: single-phase vs. cross-phase tasks, structured vs. unstructured boards, and isolated vs. shared plenary synthesis.<br>Please acknowledge in the discussion that the study evaluates two distinct "pedagogical packages" rather than isolating the single effect of phase-specific task allocation.	Thank you for this insightful observation. We agree that the intervention formats include several interconnected pedagogical features and that the study should not be interpreted as isolating the effect of phase-specific task allocation alone. In response, we have revised the intervention descriptions to improve alignment between the two formats. Both formats now involve small groups of 5-6 students, facilitator and timekeeper roles, identification of a shared priority action, access to the Green Theatre Checklist, and a plenary presentation of a group-generated product. We have also added a statement to the discussion acknowledging that the comparison is best understood as an evaluation of two educational or pedagogical packages, and that any differences observed between study arms should be interpreted as reflecting the combined influence of multiple pedagogical components rather than a single design feature.
Intervention Fidelity - Fidelity is stated as a key feasibility metric, yet the observation guides and adherence monitoring processes are barely	Thank you for this important comment. We agree that assessment of intervention fidelity require further detail. Fidelity will be monitored using a structured observation

described. Outline whether observers will use a structured checklist, what constitutes a protocol deviation during seminar delivery, and whether facilitators receive a standardized briefing/manual.	protocol completed during seminar delivery. The protocol includes checklist-based assessment of adherence to the planned intervention components and an overall fidelity rating (high, moderate, or low). The observation protocol is referred to in the manuscript under Data Collection and Tools and as Supplemental Material.
**Minor points**	
Terminology: Standardise the terminology used for the intervention arms across the text, tables, and appendices (currently alternating between "phase-based," "phase-structured," "cross-phase," and "domain-based")	Thank you for this helpful suggestion. We agree that the use of multiple terms to describe the intervention arms may create unnecessary ambiguity. To improve clarity and consistency, we have standardised the terminology throughout the manuscript, tables, figures, and supplementary materials. The intervention arms are now referred to consistently as the phase-specific format (Format A) and the cross-phase format (Format B).
Green Theatre Checklist Alignment: Clarify the mapping between the four checklist domains (which include anaesthesia) and the three perioperative phases (pre-, intra-, post-op) assigned to the nursing students.	Thank you for this helpful observation. We recognise that the relationship between the Green Theatre Checklist and the perioperative phases was insufficiently explained.<br>We have revised the manuscript to clarify that the Green Theatre Checklist is used as a preparatory resource and source of inspiration for students' discussions rather than as a framework for allocating tasks across the intervention arms. The checklist is intended to stimulate reflection on sustainability practices within the operating theatre context and to provide examples of potential sustainability initiatives.<br>We have also clarified that the anaesthesia domain of the Green Theatre Checklist is not included in the present study because the participants are operating theatre nursing students. However, the anaesthesia domain may be highly relevant in future interprofessional versions of the seminar involving nurse anaesthetists, anaesthesiologists, and other members of the perioperative team.<br>Corresponding clarifications have been added to the intervention description.<br><br>There is no intended one-to-one mapping. The checklist functions as a preparatory and inspirational resource, whereas perioperative phases are used to structure the learning activity.
COIL-I Response Scale: Verify the Likert scale phrasing in the adapted COIL-I, ensuring balanced positive/negative anchors across the options.	Thank you for this comment. We have verified the response scale wording against the original COIL-I instrument. The original response scale and response anchors have been retained unchanged, and no modifications have been made to the scoring procedures. We have clarified this in the manuscript and now explicitly state that the original response scale was preserved to maintain fidelity to the instrument and support comparability with previous applications.

Grammar & Mechanics: Correct minor phrasing issues throughout the manuscript (e.g., "Evidence indicate" → "Evidence indicates"; "departments implements" → "departments implement"; ensure proper possessive apostrophes in "participants' responses").	Thank you for highlighting these issues. We have carefully reviewed the manuscript and revised the specific examples including "Evidence indicates," and "participants' responses." We have also corrected similar minor grammatical and phrasing issues throughout the manuscript to improve linguistic accuracy and readability.
Study Dates: Insert the projected calendar dates for recruitment, seminar delivery, and follow-up data collection to meet standard reporting requirements.	Thank you for this suggestion. We have revised the manuscript to include the study dates for participant recruitment, seminar delivery, immediate post-seminar data collection, and one-week follow-up data collection in the Methods section and participant timeline and SPIRIT.

VERSION 2 - REVIEW

Reviewer **1**

Name **Berger-Estilita, Joana**

Affiliation **Inselspital University Hospital Bern, Department of Anaesthesiology and Pain Medicine**

Date **13-Oct-2026**

COI

The revised manuscript presents a mixed-methods randomised pilot protocol evaluating two pedagogically distinct formats for international collaborative learning in specialist nursing education. The authors have responded constructively to the earlier concerns. The feasibility aims and outcomes are clearer, the intervention and allocation procedures are better described, and the traffic-light progression criteria strengthen the protocol.

I have no major concerns that would prevent publication. I recommend minor revision to address the following points:

- The Methods state that baseline and follow-up questionnaires will be anonymous, cannot be linked at the individual level, and will be analysed as unpaired aggregate data. Please ensure that this limitation is stated consistently wherever the questionnaire analyses are described, and that the protocol does not imply that it can measure individual-level changes in attitudes. Aggregate differences between time points should be interpreted descriptively.

- The criteria for "sufficient breadth and depth" of qualitative data and for the coding framework's suitability are necessarily judgement-based. Please clarify briefly how these judgements will be made—for example, who will assess them and at what point—so that the progression decision is as transparent as possible.

These are clarifications rather than concerns about the overall design. The manuscript is substantially improved and appears suitable for publication once these points are addressed.

VERSION 2 - AUTHOR RESPONSE

Reviewer comments

I recommend minor revision to address the following points:	Author reply
- The Methods state that baseline and follow-up questionnaires will be anonymous, cannot be linked at the individual level, and will be analysed as unpaired aggregate data. Please ensure that this limitation is stated consistently wherever the questionnaire analyses are described, and that the protocol does not imply that it can measure individual-level changes in attitudes. Aggregate differences between time points should be interpreted descriptively.	We thank the reviewer for this important observation. We have clarified throughout the manuscript that baseline and follow-up questionnaire data will be analysed as independent aggregate datasets because responses are anonymous and cannot be linked at the individual level. We have further clarified that any differences between time points will be interpreted descriptively at the group level only and not as evidence of individual-level changes in attitudes. Clarifying statements have been added to the Secondary Outcomes section and the Strengths and Limitations section of the Discussion. In addition, to better reflect the exploratory and feasibility-oriented nature of the study, we have revised the title from "Evaluating learning formats in international collaborative nursing education on professional agency in perioperative sustainability: protocol for a mixed-method randomised pilot study" to "A mixed-method randomised pilot study of learning formats in international collaborative nursing education for professional agency in perioperative sustainability: study protocol."
- The criteria for "sufficient breadth and depth" of qualitative data and for the coding framework's suitability are necessarily judgement-based. Please clarify briefly how these judgements will be made—for example, who will assess them and at what point—so that the progression decision is as transparent as possible.	We thank the reviewer for this helpful suggestion. We agree that the assessment of qualitative data richness and coding framework suitability involves researcher judgement. We have therefore clarified that these assessments will be conducted by the qualitative research team following completion of data collection and preliminary qualitative analysis.<br>The manuscript now specifies that the assessments will be conducted collaboratively by MC, KB, AF and SOD following completion of data collection and preliminary qualitative analysis. Decisions will be reached through discussion and documented as part of the progression assessment process. This clarification has been added under the heading "Decision-making and progression criteria".
