# Supplementary material for: Evaluating learning formats in international collaborative nursing education on professional agency in perioperative sustainability: protocol for a mixed-methods, randomised pilot study
Source: BMJ Open. 2026 Nov 11;16(11):e124998. doi: 10.1136/bmjopen-2026-124998 (PMC13756281; doi:10.1136/bmjopen-2026-124998)
Supplement: Supplementary file 5 [file bmjopen-16-11-s004.docx]

**Supplemental material Coding Framework**

Developed by the authors, this coding framework integrates sustainability education (Redman & Wiek, 2021) and professional agency (Biesta, Priestley, & Robinson, 2015) to conceptualise support for operating theatre nursing students’ development of ethically informed and contextually responsive sustainable practice.

| **Scheme for coding professional agency for sustainable perioperative practice** | | |
| --- | --- | --- |
| **Dimension** | **Coding indicators** | **Definition** (how the student…) |
| **Past-oriented and professional norms** | “in my clinical placement…” “it is common practice…” “theatre nurses usually…” | Draws on past experience, professional norms, and established ways of working |
| **Future-oriented intentions and actions** | “we could reduce…” “I would prioritise…” “this should be implemented…” | Imagines change and propose actions |
| **Judgement in context** | “but this depends on…” “it might be difficult because…” “in practice…” | Weighs opportunities against constraints and judges what is realistic in practice |
| **Ethical values in sustainability reasoning** | “we should not waste…” “it is important for the environment…” “this is not ethically justified…” | Reflects on what is right or wrong and weighs sustainability values |
| **Agency in interaction and collaboration** | “we need to discuss with surgeons…” “this requires team collaboration…” “different roles…” | Considers actions in relation to others and engages with interprofessional collaboration |
| **Feasible transformation** | Specific actions priorities step-by-step solutions | Makes strategies concrete and links ideas to practice |

Redman, A., & Wiek, A. (2021). Competencies for advancing transformations towards sustainability. Frontiers in Education, 6, 785163. <https://doi.org/10.3389/feduc.2021.785163>

Biesta, G., Priestley, M., & Robinson, S. (2015). The role of beliefs in teacher agency. Teachers and Teaching, 21(6), 624–640. <https://doi.org/10.1080/13540602.2015.1044325>
